# Local cross-border disease surveillance and control: experiences from the Mekong Basin

**DOI:** 10.1186/s13104-015-1047-6

**Published:** 2015-03-21

**Authors:** Melinda Moore, David J Dausey

**Affiliations:** Health Unit, RAND Corporation, Arlington, VA USA; School of Health Professions and Public Health, Mercyhurst University, Erie, PA USA

**Keywords:** Surveillance, Regional, Sub-regional, Network, Cross-border, Cooperation, Mekong, International Health Regulations, Public health, Global health

## Abstract

**Background:**

The Mekong Basin Disease Surveillance cooperation (MBDS) is one of several sub-regional disease surveillance networks that have emerged in recent years as an approach to transnational cooperation for infectious disease prevention and control. Since 2003 MBDS has pioneered a unique model for local cross-border cooperation. This study examines stakeholders’ perspectives of these MBDS experiences, based on a survey of local managers and semi-structured interviews with MBDS leaders and the central coordinator.

**Results:**

Fifteen managers from 12 of 20 paired cross-border sites completed a written survey. They all monitor most or all of the 17 diseases agreed upon for MBDS surveillance information sharing. Fourteen agreed or strongly agreed with statements about the core MBDS values of cooperation, mutual trust, and transparency, and their own contributions to national and regional disease control (average score of 4.4 of 5.0). Respondents felt they implemented well to very well activities related to surveillance reporting (average scores 3.4 to 3.9 of 4.0), using computers for their work (3.9/4.0), and using surveillance data for action (3.8/4.0). Respondents reported that they did worst in implementing research (2.1/4.0) and somewhat poorly for local laboratory testing (2.9/4.0) and local coordination with cross-border counterparts (2.9/4.0), although all 15 maintain a list with contact information for these counterparts and many know their counterparts. Implementation of specified activities within their collective regional action plan was uneven across the cross-border sites. Most respondents reported positive lessons learned about local cooperation, information sharing and joint problem solving, based on trusting relationships with their cross-border counterparts. They recommend expansion of cross-border sites within MBDS and consideration of the cross-border cooperation model by other sub-regional networks.

**Conclusions:**

MBDS has over a decade of experience with its model of local cross-border cooperation in disease surveillance and control. Frontline managers have documented success with this model, strongly support it and recommend its expansion within and beyond the MBDS network. The MBDS cross-border cooperation model is standing the test of time as a solid approach to building and sustaining the public health capabilities needed for disease surveillance and control from the local to national and global levels.

**Electronic supplementary material:**

The online version of this article (doi:10.1186/s13104-015-1047-6) contains supplementary material, which is available to authorized users.

## Background

**Figure 1:**
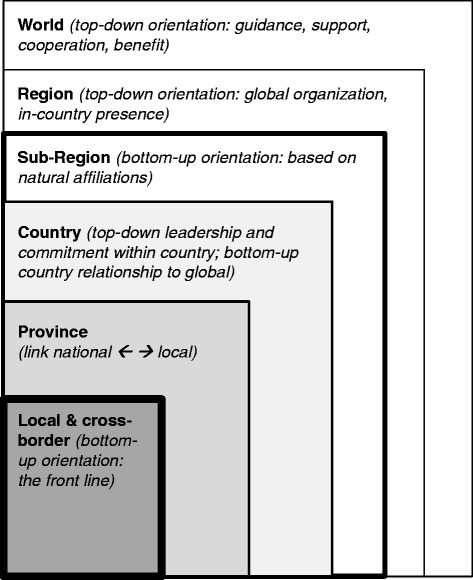
**Local officials are at the front lines of public health.**

In today’s globalized world, infectious disease threats have become transnational in nature and therefore require effective transnational approaches for detection, response and prevention [1-5]. Through the World Health Organization’s (WHO) International Health Regulations (IHR), nearly all countries around the world have committed to develop and maintain core public health capacities needed to detect, diagnose, report and respond to public health threat [6]. Countries that can do so have committed to help other countries develop their core capacities. However, the foundation of transnational detection and response begins locally, where diseases occur. Local officials are on the front lines of public health surveillance and response (Figure 1).

Self-organized sub-regional disease surveillance networks have emerged in recent years as a model of transnational public health cooperation for disease surveillance and control [5,7-17]. Such networks have a bottom-up orientation in the sense that they are self-organized affiliations rather than assigned ones. They contrast with regions organized in a top-down fashion, such as those designated by WHO.

The Mekong Basin Disease Surveillance (MBDS) cooperation is one of the longest standing sub-regional disease surveillance networks [7,9]. MBDS includes Cambodia, Lao PDR, Myanmar, Thailand, Vietnam and the Yunnan and Guangxi provinces of China. Organized initially in 1999 and formalizing its cooperation in 2001, MBDS has country level managers and a coordinator’s office located in Bangkok, Thailand. MBDS stakeholders organized their activities based on multi-year action plans generated by MBDS members and leadership. The plan in place at the time of this study was for 2011–2016 and specified seven strategic areas for national action and sub-regional cooperation: cross-border (XB) cooperation; strengthening the animal-human health interface and community surveillance; epidemiology capacity building; laboratory capacity building, information and communications capacity building; risk communications; and policy research [18]. Through its XB strategy, MBDS has pioneered a specific type of model for cooperation: a multi-country networked system of local XB sites to cooperate directly on disease surveillance, information sharing and joint investigation across local international borders [9].

The term “cross-border” in the context of public health and disease is commonly used as a synonym for “transnational” [19-22] rather than referring literally to local collaborations across international borders. Examples of the former focus on descriptions of cross-border disease threats [7,19-21]. Examples of the latter focus on local cross-border surveillance cooperation [7-9,23], “cross-border sharing of human resources and expertise [7], “[stamping] out the cross-border [dengue] outbreak” [7], “cross-border response teams [7], cross-border communications [8], and meetings at cross-border sites [8,9,23]. Some uses of the term are more ambiguous as to whether such actions as cross-border population movements [7,14,23], cross-border trade [7], cross-border collaboration [7], and cross-border communications [7,8] refer to broad or more local transnational concepts, or both.

Between 2003 and 2012, MBDS established 25 XB demonstration sites. These provide a unique, “bottom-up,” complementary approach to local, national and transnational disease surveillance and control. This report focuses on the MBDS experience with local XB cooperation in disease surveillance and control. It describes insights about such cooperation as seen from various perspectives. These include the perspectives of local XB site managers, who are responsible for implementing and managing activities at their site; MBDS country leaders, who are responsible for coordinating MBDS efforts in their country and contributing to decision making through the MBDS Executive Board; and the MBDS central coordinator, who is responsible for coordinating efforts across all MBDS countries. The study reported here examines the following research questions:

1. How well do local XB health authorities understand their role in national surveillance, MBDS networking, and the WHO International Health Regulations?
2. Which areas specified for MBDS cooperation are current public health priorities at the local level?
3. How well have MBDS strategic priorities and activities been implemented at XB sites?
4. To what extent is surveillance data/information shared and used locally?
5. What aspects of surveillance are working well and less well at these sites?
6. What was the sequence of activities in developing the XB sites?
7. What activities are viewed as the most important or valuable at XB sites?
8. What lessons have been learned from XB cooperation, and what advice could be offered to others?
9. What are the prospects for sustainability of XB cooperation, including enabling factors and barriers?

Insights from this study not only help to improve MBDS’s own programming but also are valuable to inform cooperation in other disease surveillance networks that span international borders or require communication and coordination across different agencies and organizations. In addition, public health workers broadly focused on disease surveillance may find the results of the study helpful as they consider collaborative approaches to disease surveillance.

## Methods

The study was carried out from January 2012 to January 2013. During this time period, 20 of the 25 designated MBDS XB demonstration sites had one or both sides operational. After consultation with leaders in the MBDS member countries, the coordinator requested in writing that RAND’s human subjects protection committee carry out the ethical review on their behalf. Therefore, RAND’s human subjects protection committee approved the study on behalf of both RAND and MBDS. Data collection included a written survey during 2012 and semi-structured interviews in early 2013, both of which included verbal informed consent that had been approved by RAND’s ethical review committee.

The survey questionnaire was presented and completed in English by local XB site managers. It included both open-ended questions and closed-ended questions with checked, binary (yes or no), or scaled (1 to 4 or 1 to 5) responses (see Additional file 1). The targeted survey sample included all MBDS XB sites, including those pairs operating on both sides of the border and pairs where only one side of the border was operational. The MBDS central coordinator worked with MBDS country leaders to ensure that representatives from as many sites as possible had an opportunity to complete the survey. Survey information was collected via written questionnaire and transmitted electronically to the study team. A total of 15 XB local site managers in five of the six MBDS countries completed surveys. These managers represented 12 of the 20 different XB sites active at the time of the survey (Table 1). These included paired forms from both sides of four XB sites (one site in Lao PDR is part of two different pairs) and single forms from eight additional sites. Responses to the survey’s closed-ended questions were tallied and averaged. Responses to the open-ended questions were extracted, arrayed, and either listed or summarized.

**Table 1:** **MBDS cross-border sites and source of completed survey forms**

**#**	**Status**	**Year started**	**# forms received**	**Cambodia**	**China**	**Laos**	**Myanmar**	**Thailand**	**Vietnam**
1	●	2003	1			*Savannakhet (a)*		**Mukdahan**	
2	●	2003	0			*Savannakhet (b)*			*Quang Tri*
3	●	2003	2	**Stung Treng**		**Champasak (a)**			
4	●	2003	0		*Mengla (Yunnan)*	*Luang Namtha*			
5	●	2008	0			*Bo Kaeo*		*Chiang Rai (a)*	
6	●	2008	2	**Banteay Mean Chey**				**Sakaeo**	
7	●	2008	1	**Takaeo**					*An Giang*
8	●	2008	1	**Kampot**					*Kien Giang*
9	●	2008	1		*Luchun (Yunnan)*				**Lai Chau**
10	●	2008	1		*Ping Xiang (Guangxi)*				**Lang Son**
11	●	2009	1			*Borikhamxay*			**Ha Tinh**
12	●	2009	0			*Vientiane*		*Nongkhai*	
13	●	2009	0			*Sayabury*		*Nan*	
14	●	2011	2	**Koh Kong**				**Trat**	
15	Ө	2011	0		*Dong Xing (proposed)*				*Guang Ninh*
16	Ө	2011	0	*Kampong Cham*					*Tay Ninh (a)*
17	Ө	(2012)	2			**Champasak (b)**		**Ubon Ratchathani**	
18	Ө	(2012)	1	**Svay Rieng**					*Tay Ninh (b)*
19	Ө	(2012)	1				**Myawaddy**	*Mae Sot*	
20	Ө	(2012)	0		*Dong Xing*				*Mong Cai*
21	O	(2012)	0				*Tachilake*	*Chiang Rai (b)*	
22	O	(2012)	0	*Battambang & Pailin*				*Chanthaburi*	
23	O	(2012)	0			*Khammouane*		*Nakorn Phanom*	
24	O	(2012)	0			*Khammouane*			*Quang Binh*
25	O	(2012)	0				*Kawthaung*	*Ranong*	

Legend: O = Identified, not operational either side; Ө = Ready (coordinator, plan/TOR, or just 1 side operational); ● = Fully operational; **bold** = form received; *italic* = form not received; (a) and (b) refer to single sites that are part of more than one XB pair.

In addition, the RAND study team completed face-to-face interviews with two MBDS country leaders and the MBDS central coordinator in early 2013. These discussions explored how and why certain program elements were more or less successful than others, to help inform replication or new approaches in the future. As with the open-ended survey questions, responses were extracted, arrayed and either listed or summarized.

## Results and discussion

All respondents indicated that they monitor at least 14 of the 17 diseases or conditions agreed upon for MBDS surveillance information sharing (acute flaccid paralysis, avian influenza, Chikungunya fever, cholera, dengue, diphtheria, encephalitis, human immunodeficiency virus [HIV], leptospirosis, malaria, measles, meningitis, pneumonia, severe acute respiratory syndrome [SARS], tetanus, tuberculosis, and typhoid); ten indicated that they monitor all 17 of these. Overall and not surprisingly, survey respondents from the 15 sites were most familiar with their own country’s surveillance system and their local MBDS XB cooperation (Table 2). They reported being well aware of the WHO IHR in general but they were less aware of specific elements of the IHR. They were more aware of the MBDS central coordinator and his office, which communicates relatively regularly with the XB sites, than with the MBDS country leaders. Respondents whose counterparts across the border did not complete the survey (reflected as “Singles” in Table 2) were more aware of nearly all aspects of MBDS, their country’s surveillance, and the IHR, compared to respondents whose XB counterparts did complete the survey (reflected as “Pairs” in Table 2). Familiarity with MBDS, national surveillance and the IHR was somewhat lower for respondents from the four sites in Thailand (average score of 2.8 of 5.0) compared to those from the six sites in Cambodia (4.1/5.0) or the three sites in Vietnam (4.0/5.0). No information on the surveys or in the interviews pointed to the reasons for these differences.

**Table 2:** **Respondent awareness/familiarity with MBDS**

**Description (Item)**	**Average score**					
	***(Scale: 1 not aware to 5 very aware)***					
	**ALL**	**Pairs**	**Singles**	**Cambodia**	**Thailand**	**Vietnam**
Number of sites	15	7	8	6	4	3
MBDS - general	3.4	2.9	3.9	3.2	2.8	4.0
MBDS - Executive board	3.5	2.4	4.5	3.7	2.5	4.3
MBDS - Country coordinator	3.9	3.3	4.4	4.2	2.8	4.3
MBDS Coordinator/coordinating office	4.0	3.4	4.5	4.7	2.8	4.3
MBDS Action plan and strategies	3.8	3.4	4.1	3.7	3.0	4.3
Cross-border (XB) cooperation	4.1	3.9	4.4	4.7	3.5	4.0
Your country’s surveillance program	4.3	4.3	4.4	4.5	3.5	4.7
IHR	4.0	4.1	3.9	4.5	3.3	3.3
IHR reporting requirements	3.5	3.0	3.9	4.0	2.3	3.7
IHR core capacities	3.3	2.9	3.8	4.0	2.0	3.3
*Total*	3.8	3.4	4.2	4.1	2.8	4.0

Respondents were asked which of 11 specific surveillance-related activities or capacities are priorities for their country, MBDS, and/or the WHO IHR:

- Infectious disease surveillance
- Timely surveillance reporting
- Using surveillance information for action
- Public health capacity building
- Laboratory capacity
- Epidemiology capacity building
- Risk communications
- Communications technology capacity
- Surveillance at points of entry
- Public health emergencies of international concern
- Coordination of animal and human health

The vast majority of respondents (13; 87%) indicated that all 11 of these are important to their country (overall average 10.8/11); 10 (67%) indicated that all 11 are important for MBDS (9.9/11.0); and 6 (40%) indicated that all 11 are important to the IHR (8.9/11). These findings are consistent with respondents’ higher familiarity with their own national surveillance system and MBDS cooperation than with details of the WHO IHR, though general familiarity with the WHO IHR is relatively high (average score for awareness/familiarity of 4.0 of 5.0).

Nearly all respondents (14; 93%) agreed or strongly agreed with all statements about the importance of MBDS cooperation, trust, and transparency; the consistency of MBDS with the country’s own surveillance and response system; the contribution of their own work to the country’s surveillance system; and the importance of exercises and drills (Table 3). A small minority of respondents (3; 20%) was neutral about the statement that their work serves the MBDS system. One respondent in Thailand appeared to be an outlier and disagreed or strongly disagreed with all of these statements.

**Table 3:** **Values and context as reported by respondents**

**Description (Item)**	**Average score**					
	***(Scale: 1 strongly disagree to 5 strongly agree)***					
	**ALL**	**Pairs**	**Singles**	**Cambodia**	**Thailand**	**Vietnam**
Number of sites	15	7	8	6	4	3
MBDS cooperation is important	4.4	4.3	4.6	4.8	3.5	4.5
MBDS is a pioneer for cooperation	4.5	4.7	4.3	4.5	3.5	4.3
Mutual trust is important in MBDS	4.5	4.1	4.8	4.5	3.8	5.0
Transparency is important in MBDS	4.5	4.1	4.9	4.8	3.5	5.0
Your work serves MBDS system	4.1	3.7	4.5	4.3	3.8	4.3
Your work serves country system	4.4	4.1	4.6	4.5	3.8	4.7
MBDS is consistent with country’s surveillance and response system	4.3	3.9	4.6	4.3	4.0	4.7
Exercises (tabletops, simulations) and drills are important	4.5	4.1	4.9	4.7	4.0	4.7
*Total*	4.4	4.1	4.6	4.6	3.7	4.7

Most respondents consider that they implement moderately to very well most of the general activities associated with MBDS (Table 4). These include reporting surveillance data to their country surveillance system (average score 3.9 of 4.0), their XB partner (3.4/4.0) and the MBDS Coordinator (3.5/4.0); using their surveillance data for local action (3.8/4.0); responding locally to disease outbreaks (3.7/4.0); coordinating human and animal health (3.2/4.0); conducting community-based surveillance (3.4/4.0); using computers in their work (3.9/4.0); and carrying out risk communications (3.4/4.0). Respondents felt that they conducted joint outbreak investigations (2.9/4.0) and local laboratory testing (2.9/4.0) somewhat more poorly, and conducted applied or other research poorly or not at all (2.1/4.0). (A possible explanation for the low perceived quality of policy research implementation is that XB sites would not necessarily initiate, carry out or even be aware of such research.) Respondents at sites from which both XB partners completed the survey (“Pairs”) reported better coordination (3.1/4.0) and surveillance reporting (3.7/4.0) to their XB partner compared to respondents whose XB counterpart did not complete the survey (“Singles”, 2.8/4.0 and 3.1/4.0, respectively). Respondents from the six sites in Cambodia felt that their lab testing (2.5/4.0), joint outbreak investigations (2.3/4.0), and coordination of human and animal health (2.8/4.0) were more poorly implemented than respondents from Thailand (3.3/4.0, 3.0/4.0, 3.5/4.0, respectively) or Vietnam (3.3/4.0, 4.0/4.0, 3.3/4.0, respectively). Nearly all respondents look at and report their surveillance data, but somewhat fewer analyze or use these data on a regular basis (Table 5).

**Table 4:** **Respondent perception of quality of local implementation**

**Description (Item)**	**Average score**					
	***(Scale: 1 do not implement to 4 implement very well)***					
	**ALL**	**Pairs**	**Singles**	**Cambodia**	**Thailand**	**Vietnam**
Number of sites	15	7	8	6	4	3
Coordinate, talk, and/or meet with XB counterparts	2.9	3.1	2.8	2.7	3.5	2.7
Report surveillance data to country system	3.9	4.0	3.9	4.0	3.8	4.0
Report surveillance data to XB partner	3.4	3.7	3.1	3.8	3.5	2.7
Report surveillance data to MBDS Coordinator	3.5	3.7	3.4	3.8	3.5	3.3
Use your surveillance info for action	3.8	3.9	3.8	4.0	3.8	3.7
Local outbreak response/investigation	3.7	3.9	3.6	3.8	3.5	3.7
Joint XB outbreak response/investigation	2.9	2.4	3.3	2.3	3.0	4.0
Coordinate human-animal health	3.2	3.1	3.3	2.8	3.5	3.3
Conduct community-based surveillance	3.4	3.1	3.6	3.2	3.5	3.7
Build and use epidemiology capacity	3.3	3.1	3.5	3.0	3.5	3.7
Conduct lab testing for priority diseases	2.9	2.7	3.1	2.5	3.3	3.3
Use computers for your routine work	3.9	3.7	4.0	4.0	3.8	4.0
Conduct risk communications	3.4	3.1	3.6	3.3	3.3	3.7
Conduct applied or other research	2.1	1.4	2.8	2.0	2.0	2.7
*Total*	3.3	3.2	3.4	3.2	3.4	3.5

**Table 5:** **Use of surveillance data by respondents**

**Description (Item)**	**Percentage of sites reporting “Yes”**					
	**ALL**	**Pairs**	**Singles**	**Cambodia**	**Thailand**	**Vietnam**
Number of sites	15	7	8	6	4	3
Look at data	93%	100%	88%	100%	100%	67%
Analyze data	87%	86%	88%	83%	75%	100%
Use data	87%	86%	88%	83%	100%	67%
Report data	93%	100%	88%	100%	75%	100%
*Average (of 4 possible actions)*	3.6	3.7	3.5	3.7	3.5	3.3

The XB managers were asked to indicate whether they implement a number of specific activities that are relevant to XB sites, i.e., linked to the first six key strategies in the MBDS Action Plan for 2011–2016. (In contrast, the seventh strategy, policy research, does not specifically involve activities at all XB sites.) All respondents reported that they maintain a list of contact information for their XB counterparts (Table 6). Nearly all have a basic package of activities for their site and share surveillance information as required (for agreed-upon diseases at specified frequencies). More than three-fourths have ever participated in a joint XB outbreak investigation; slightly more than half have participated in at least one meeting with their XB counterpart or had a supervisory visit during the preceding six months. Of the six MBDS strategies reflected in the table, implementation of specific activities associated with epidemiology capacity (present all sites) and XB cooperation (average 5.3 of 7 different XB-specific activities implemented) was most common. Activities associated with information and communications technology capacity (average 3.6 of 4 different activities in this area), animal-human health interface and community surveillance (average 4.5 of 7 different activities), risk communications (average 1.1 of 2 activities), or laboratory capacity (average 1.6 of 3 different activities) were less common. The fifteen sites implement an average 17.0 of the total 24 activities. The six Cambodian sites reported implementing more activities (average 18.7/24) than the four sites in Thailand (average 16.0/24) or the two sites reporting from Vietnam (average 15.0/24).

**Table 6:** **Implementation of specific activities at MBDS XB sites**

**Description (Item)**	**Percentage reporting “Yes”**					
	**ALL**	**Pairs**	**Singles**	**Cambodia**	**Thailand**	**Vietnam**
Number of sites	15	7	8	6	4	2*
*Cross-border (XB) Cooperation*						
Maintain list of contact info for XB counterparts	100%	100%	100%	100%	100%	100%
Have a basic package of activities for your site	86%	86%	86%	83%	75%	100%
Shared surveillance information as required	93%	100%	86%	100%	100%	100%
Participated in at least one meeting with another XB site in the past 6 months	57%	43%	71%	83%	50%	50%
Participated in at least one supervisory visit in the last 6 months	57%	57%	57%	67%	25%	100%
Ever participated in joint outbreak investigation	79%	71%	86%	67%	75%	100%
Participated in at least one outbreak investigation, TTX or drill past 12 months	57%	43%	71%	50%	50%	100%
*Average number (of 7 possible)*	5.3	5.0	5.6	5.5	4.8	6.5
*Animal-human interface and community-based surveillance*						
Maintain list of priority zoonotic diseases	86%	86%	86%	83%	100%	50%
Maintain a list of contact information for local animal & human health counterparts	79%	86%	71%	83%	75%	50%
Participated in outbreak investigation, TTX or drill that at addressed the interface between animal and human health in the past 12 months	57%	43%	71%	50%	50%	100%
Regularly share surveillance reports between animal and human health sectors	64%	57%	71%	67%	25%	100%
Have list of suspected diseases or events to report via community-based surveillance	71%	57%	86%	67%	50%	100%
Have tested (pilot) or implemented community-based surveillance past 6 months	36%	14%	57%	50%	0%	100%
Community-based surveillance fully operational at site	57%	29%	86%	83%	25%	50%
*Average number (of 7 possible)*	4.5	3.7	5.3	4.8	3.3	5.5
*Human resource/epidemiology capacity*						
At least 1 person at site has participated in short- or long-term epidemiology course	100%	100%	100%	100%	100%	100%
*ICT capacity*						
XB site has ICT hardware/software installed, including updates.	93%	100%	86%	100%	100%	33%
XB site has received ICT training, including updates as needed.	79%	86%	71%	100%	100%	33%
XB site has access to ICT support when needed.	93%	100%	86%	100%	100%	33%
XB site routinely uses ICT for surveillance.	93%	100%	86%	100%	100%	33%
*Average number (of 4 possible)*	3.6	3.9	3.3	4.0	4.0	2.0
*Laboratory capacity*						
Site has laboratory for detecting/diagnosing at least 1 priority disease	71%	86%	57%	83%	75%	0%
Site has timely access to lab testing for all priority diseases	36%	57%	14%	33%	75%	0%
Laboratory at or serving your site participated in proficiency testing past 12 months	50%	43%	57%	67%	50%	0%
*Average number (of 3 possible)*	1.6	1.9	1.3	1.8	2.0	0.0
*Risk communications (RC)*						
At least 1 person at your site has received risk communications training	64%	71%	57%	83%	50%	0%
Your site has used (in a real situation) or tested (via exercise) RC past 12 months	43%	43%	43%	67%	50%	0%
*Average number (of 2 possible)*	1.1	1.1	1.0	1.5	1.0	0.0
*Overall average number (of 24 possible)*	17.0	16.6	17.4	18.7	16.0	15.0

*Only 2 of the 3 sites in Vietnam reported this information.

Respondents commented on the first activities needed to start up an XB site, which XB activities have been most valuable, and lessons they have learned about XB cooperation. Nearly all reported that initial activities included meetings with national or provincial authorities as well as their XB counterparts, orientation and training, and sharing surveillance information with XB partners. Nearly all also reported that the most valuable activities were sharing information, meeting regularly, and conducting joint outbreak investigations with XB counterparts. Most reported positive lessons learned about local cooperation, information sharing and joint problem solving, all based on trust, mutual respect and good relationships with XB counterparts. Respondents also offered advice to future MBDS XB sites or to other countries or networks that may establish similar sites. They recognized the importance of initial local and cross-border orientation, regular meetings with XB counterparts to maintain good relationships, an established agreement at the XB site, and openness and timeliness in sharing surveillance information across borders. Based on their experiences, they recommend expansion of the XB model more broadly across MBDS and feel that it is a worthwhile model for other sub-regional networks to consider.

Nearly all respondents commented on the aspects of surveillance that are working well at their site. Responses varied, with no consensus themes. Some of the reported well-functioning elements included both routine case-based and community event-based reporting, coordination from national to local level, and the availability of specific guidelines and communications technologies for surveillance reporting. Several respondents also commented on aspects of surveillance that are not working well. These include village level community surveillance (functioning well at some sites but not well at others), lack of local laboratory testing availability, and limited budget and staff motivation or participation. Most respondents explicitly noted the importance of the sustainability of their XB cooperation. They were at least moderately confident that they could sustain their efforts if they could maintain their good relationships with XB counterparts and receive sufficient technical and especially financial support.

Interviews with the two senior country level MBDS managers and the MBDS central coordinator reinforced and expanded upon the insights provided by the XB survey respondents. They all recognized the strengths and weaknesses of MBDS cooperation over time. Strengths include acknowledgement of the XB model as a good foundation for building trust, sharing surveillance information, conducting joint outbreak investigations, and collaborating more broadly. The major weakness is that implementation and capacity are uneven across countries and local XB sites. More specifically, these leaders identified the need to more extensively and actively use surveillance information for action (rather than merely sharing it) and enhance laboratory capacity across all countries and out to the XB level. Nonetheless, after a decade of experience in working together, they feel that the MBDS cooperation has been successful and the MBDS XB model has contributed importantly to both local disease control and compliance with the IHR. They feel the XB model should be strengthened and expanded—by strengthening local human, laboratory and communications technologies and expanding to more counterpart XB sites along the expansive MBDS borders. One manager noted the political and practical importance of local XB cooperation in areas beyond simply disease surveillance. He further noted that sustainability will depend more on governments integrating MBDS-related activities into their routine programming and providing ongoing financial support to do so, rather than depending on external funding into perpetuity.

## Conclusions

MBDS has more than a decade of experience with its model of local cross-border cooperation in disease surveillance and control. Frontline XB managers strongly support this model and hope it can be sustained and expanded, both within and beyond MBDS. They especially noted the importance of relationships built on trust, which in turn enhance disease surveillance and control at local transborder sites. Senior MBDS officials validated these views, and recent commentaries also support local cross-border cooperation as a promising pathway for the future [16,24]. The MBDS Action Plan spells out seven key strategies, of which six are directly and strongly relevant to all XB sites and hence were the major focus of our examination, as reported here. Survey respondents indicated that XB cooperation and epidemiology capacity are the strongest in underpinning current MBDS cooperation; some key capacities remain uneven across the XB sites, especially laboratory and communications technologies/capacities. The challenges to public health surveillance and networking have been described [5,25]. Building and sustaining a full set of critical public health surveillance capacities across all MBDS XB sites will indeed be a challenge for the future. However, the MBDS XB model is standing the test of time as a solid approach to building and sustaining the public health capabilities needed into the future for disease surveillance and control from the local to national and global level.

## Additional file

Additional file 1:
**Cross-Border Disease Surveillance – Site Manager Questionnaire.**

## Abbreviations

HIV: Human immunodeficiency virus
IHR: International health regulations
MBDS: Mekong basin disease surveillance
SARS: Severe acute respiratory syndrome
TOR: Terms of reference
WHO: World health organization
XB: Cross-border
