# Supplementary material for: Local cross-border disease surveillance and control: experiences from the Mekong Basin
Source: BMC Res Notes. 2015 Mar 21;8:90. doi: 10.1186/s13104-015-1047-6 (PMC4374506; doi:10.1186/s13104-015-1047-6)
Supplement: Additional file 1: — Cross-Border Disease Surveillance – Site Manager Questionnaire. [file 13104_2015_1047_MOESM1_ESM.pdf]

Date (dd-mm-yy) ☐ **XB Pair ID** ☐ **Questionnaire ID** ☐

Cross-Border Disease Surveillance

(1) XB Site Data

Country		XB: which side(s) are operational?	BOTH sides		YOUR side only		OTHER side only	

(2) MBDS and Other Basics

How aware or familiar are you with the following? *(enter response for each item-see key below)*

Description	*Response*
MBDS in general	
MBDS Executive Board	
MBDS Country Coordinator	
MBDS Coordinator/Coordinating Office	
MBDS Action Plan and strategies	
MBDS cross-border (XB) cooperation	
Your country's surveillance program	
International Health Regulations (IHR)	
IHR reporting requirements	
IHR core capacities	

Response Key:

1 = Not at all aware/familiar

2 = A little aware/familiar

3 = Somewhat aware/familiar

4 = Moderately aware/familiar

5 = Very aware/familiar

(3) Values and Context

How much do you agree or disagree with the following? *(enter response for each item-see key below)*

Description	*Response*
MBDS cooperation is important	
MBDS is a pioneer for cooperation	
Mutual trust is important in MBDS	
Transparency is important in MBDS	
Your work serves MBDS system	
Your work serves country system	
MBDS is consistent with country surveillance & response system	
Exercises (TTXs or simulations) and drills are valuable	

Response Key:

1 = Strongly disagree

2 = Disagree

3 = Neither disagree or agree

4 = Agree

5 = Strongly Agree

9 = Don't know

(4) Strategic priorities

Which are high priorities for your country, MBDS, &/or IHR? *(enter response for each-see key)*

Description	*For Country*	*For MBDS*	*For IHR*
	Response	*Response*	*Response*
Infectious disease surveillance			
Timely surveillance reporting			
Using surveillance information for action			
Public health capacity building			
Laboratory capacity			
Epidemiology capacity			
Risk communications			
Information/Communications capacity			
Surveillance at points of entry			
Public health event of international concern			
Coordination of human & animal health			

Response Key:

0 = No

1 = Yes

(5) Quality of implementation / activities

How well do you implement the following *at your site?* *(enter response for each item-see key)*

Strategy or Activity	*Response*
Coordinate, talk &/or meet with XB counterparts	
Report surveillance data to country system	
Report surveillance data to cross-border partner	
Report surveillance data to MBDS Coordinator	
Use your surveillance information for action	
LOCAL outbreak response/investigation	
JOINT (XB) outbreak response/investigation	
Coordinate human-animal health	
Conduct community-based surveillance	
Build and use epidemiology capacity	
Conduct lab testing for priority diseases	
Use computers for your routine work	
Conduct risk communications	
Conduct applied or other research	

Response Key:

1 = Do not implement

2 = Implement poorly

3 = Implement moderately well

4 = Implement very well

(6) Diseases surveillance

6.1. Indicate your response for each of the diseases below

Disease	MBDS surveillance? (no/yes)	Country surveillance? (no/yes)	Know Case Definition? (no/yes)	Reporting FREQUENCY
	Response	*Response*	*Response*	*Response*
Acute flaccid paralysis (AFP)				
Avian influenza				
Chikungunya				
Cholera				
Dengue				
Diphtheria				
Encephalitis				
HIV				
Leptospirosis				
Malaria				
Measles				
Meningitis				
Pneumonia				
SARS				
Tetanus				
Tuberculosis				
Typhoid				

Response Key:

No/Yes questions:

0 = No

1 = Yes

Frequencies:

1 = 24 hour

2 = Weekly

3 = Monthly

4 = Quarterly

6.2. What *reporting template/s* do you use for reporting surveillance data from your site?
(check ALL that apply)

Country template ☐ MBDS template ☐ WHO/IHR template ☐

6.3. What do you *personally/routinely* do with your surveillance data?
(check ALL that apply)

Look at data ☐ Analyze data ☐ Use data ☐ Report data ☐

6.4. What aspects of surveillance are *working best/well* at your site, and why?

6.5. What aspects of surveillance are *not working well* at your site, and why?

(7) Implementation of specific activities and capacity levels

Please indicate response for each of the following activities *<u>at your site</u>* *(Key: 0 = No, 1 = Yes)*

**Activity or Capacity**	***Response***
Cross-border (XB) Cooperation	
Maintain a list of contact information for XB counterparts	
Have adopted or adapted a basic package of activities for your site	
Shared surveillance information weekly, monthly and quarterly as required	
Participated in at least one meeting with another XB site in the past 6 months	
Participated in at least one supervisory visit in the last 6 months	
Ever participated in joint outbreak investigation	
Participated in at least one outbreak investigation, TTX or drill past 12 months	
Animal-human interface and community-based surveillance	
Maintain a list of priority zoonotic diseases for XB cooperation	
Maintain a list of contact information for local animal & human health counterparts	
Participated in outbreak investigation, TTX or drill that at least partially addressed the interface between animal and human health in the past 12 months	
Regularly share surveillance reports between animal and human health sectors	
Have list of suspected diseases or events to report via community-based surveillance	
Have tested (pilot) or implemented community-based surveillance past 6 months	
Community-based surveillance fully operational at site	
Human resource/epidemiology capacity	
At least 1 person at site has participated in short- or long-term epidemiology course	
ICT capacity	
XB site has ICT hardware/software installed, including updates.	
XB site has received ICT training, including updates as needed.	
XB site has access to ICT support when needed.	
XB site routinely uses ICT for surveillance.	
Laboratory capacity	
Site has laboratory for detecting/diagnosing at least 1 priority disease	
Site has timely access to lab testing for all priority diseases	
Laboratory at or serving your site participated in proficiency testing past 12 months	
Risk communications (RC)	
At least 1 person at your site has received risk communications training	
Your site has used (in a real situation) or tested (via exercise) RC past 12 months	

(8) History, opinions and suggestions

8.1. What were the *first activities* needed to start up MBDS XB work at your site?

(Possible examples include visit from national or provincial authorities, meetings within your country, meetings with XB counterparts, development of written or other agreement, training or orientation)

8.2 What are *currently the most important/valuable activities* for MBDS XB work at your site, and why are they important to you?

8.3. What are the most important lessons you have learned about XB cooperation?

8.4. What advice would you give to other districts/sites (in your country or other countries) that wish to begin XB cooperation?

8.5. With regard to sustainability of your XB activities:

8.5.1. How *important* is the sustainability of XB cooperation to you?

8.5.2. How *confident* are you that you will be able to sustain your XB cooperation?

8.5.3. What *enabling factors* would help you sustain your XB cooperation?

8.5.4. What *barriers* would impede sustainability at your XB site?

8.6. Please provide any other comments and suggestions for improving MBDS program in your cross border area?

Thank you for your participation.
